# Post-Vaccination Yellow Fever Antiserum Reduces Zika Virus in Embryoid Bodies When Placental Cells are Present

**DOI:** 10.3390/vaccines8040752

**Published:** 2020-12-11

**Authors:** Emily M. Schultz, TyAnthony J. Jones, Hannah K. Hopkins, Jingmei Zeng, Kelli L. Barr

**Affiliations:** Department of Biology, Baylor University, Waco, TX 76706, USA; Emily_Schultz1@baylor.edu (E.M.S.); Ty_jones1@baylor.edu (T.J.J.); Hannah_Hopkins2@baylor.edu (H.K.H.); Jingmei_Zeng1@baylor.edu (J.Z.)

**Keywords:** Zika virus, yellow fever virus, flavivirus, cross-reactivity, neutralization, enhancement, congenital infections, Zika congenital syndrome, stem cell

## Abstract

Zika virus (ZIKV) is a flavivirus that originated in Africa but emerged in Latin America in 2015. In this region, other flaviviruses such as Dengue (DENV), West Nile, and Yellow Fever virus (YFV) also circulate, allowing for possible antigenic cross-reactivity to impact viral infections and immune responses. Studies have found antibody-mediated enhancement between DENV and ZIKV, but the impact of YFV antibodies on ZIKV infection has not been fully explored. ZIKV infections cause congenital syndromes, such as microcephaly, necessitating further research into ZIKV vertical transmission through the placental barrier. Recent advancements in biomedical engineering have generated co-culture methods that allow for the in vitro recapitulation of the maternal–fetal interface. This study utilized a transwell assay, which was a co-culture model utilizing human placental syncytiotrophoblasts, fetal umbilical cells, and a differentiating embryoid body, to replicate the maternal–fetal axis. To determine if cross-reactive YFV vaccine antibodies impacted the pathogenesis of ZIKV across the maternal–fetal axis, syncytiotrophoblasts were inoculated with ZIKV or ZIKV incubated with YFV vaccine antisera, and the viral load was measured 72 h post-inoculation. Here, we report that BeWo and HUVEC cells were permissive to ZIKV and that the impact of YFV post-vaccination antibodies on ZIKV replication was cell line-dependent. Embryoid bodies were also permissive to ZIKV, and the presence of YFV antibodies collected 4–14 months post-vaccination reduced ZIKV infection when placental cells were present. However, when directly infected with ZIKV, the embryoid bodies displayed significantly increased viral loads in the presence of YFV antiserum taken 30 days post-vaccination. The data show that each of the cell lines and EBs have a unique response to ZIKV complexed with post-vaccination serum, suggesting there may be cell-specific mechanisms that impact congenital ZIKV infections. Since ZIKV infections can cause severe congenital syndromes, it is crucial to understand any potential enhancement or protection offered from cross-reactive, post-vaccination antibodies.

## 1. Introduction

Zika virus (ZIKV) and Yellow Fever virus (YFV) are both part of the flavivirus family, with enveloped, single-stranded, positive-sense RNA genomes. Both ZIKV and YFV are vectored by *Aedes* mosquitoes. YFV and ZIKV originated in Africa and have been found to co-circulate within the same regions of Latin America [1]. ZIKV first appeared in the Western Hemisphere in 2015 [2,3]. YFV, however, has been circulating in the Americas since the African slave trade era and is endemic in many tropical regions such as Brazil, Columbia, Venezuela, and Peru to name a few [4]. In the 1930s, a live attenuated vaccine for YFV, 17D, was developed and, in its almost 80 years of use, has proven to have a significant impact on controlling YFV outbreaks [4,5]. Multiple countries have mass vaccination programs, and some countries, where YFV is endemic, have the YFV-17D vaccine included in the national recommended childhood immunization schedule. Particularly, Bolivia, Brazil, Columbia, Ecuador, and Venezuela all recommend the vaccine to children 9–12 months of age within the entire country, not just in known endemic regions [6]. Despite these recommendations, recent surveys showed that little more than half of the population in these regions are vaccinated for YFV [7]. With the ongoing vaccination campaigns in these areas, there are a spectrum of post-vaccination YFV antibodies, some of which might enhance infections by other flaviviruses.

With many flaviviruses co-circulating in the same areas in Central and Southern America, there is the possibility of antigenic cross-reactivity, especially since some YFV-endemic areas have reported seroprevalence rates of ZIKV as high as 63% [4,8]. Antigenic cross-reactivity and antibody-mediated enhancement frequently occur between flaviviruses. Cross-reactive Dengue virus (DENV) and West Nile virus antibodies have already been shown to enhance ZIKV pathogenesis [9,10,11]. However, only limited studies have been conducted on the potential cross-reactive nature of YFV antibodies. One study, using commercial ELISA detection kits for DENV and ZIKV, found there to be minimal cross-reactivity between YFV antibodies and DENV detection, and no cross-reactivity in ZIKV detection [12,13]. While these studies were very informative, they did not represent the actual immunological landscape, as Souza et al. [12] used post-vaccination serum from 9-month-old infants, who have an undeveloped immune system, and the CDC MAC-ELISA for ZIKV was validated using a sample size of fewer than 10 individuals, of an unknown exposure history [13]. Furthermore, South America, especially Brazil, has a high incidence of measles, which can affect immunological memory in recovered persons [14].

This, however, does not indicate possible in vivo interactions, as several reports indicate that flaviviral neutralization is complex and dependent upon many factors [15]. It has also been shown that antibodies that neutralize in vitro, such as in neutralization assays, often do not neutralize in vivo, suggesting that complex immunological interactions occur for neutralization [16,17,18]. In regions where ZIKV has a high prevalence, a large portion of the population also has YFV antibodies, not only from the childhood schedule of immunizations but also from ongoing vaccination campaigns that inoculate adults and provide boosters for pregnant women, HIV-infected persons, and other immunocompromised populations [19]. With a spectrum of YFV antibodies present in this population, it is important to understand any possible cross-reactivity, antibody-mediated enhancement, or antibody-mediated neutralization.

Studies have reported that the vaccination of pregnant women with YFV occurs during vaccination campaigns [20,21]. While several studies have shown that vaccination with YFV during pregnancy is safe, the development of protective immunity is reduced, indicating that there may be increased non-neutralizing, cross-reactive antibodies [19,20,21]. Whether YFV vaccination occurs in childhood, in adulthood, or during pregnancy, cross-reactive antibodies that complex with other flaviviruses could be a source for the enhancement of infection. Furthermore, non-neutralizing antibodies have been shown to contribute significantly to antibody-dependent enhancement [22,23,24].

Since ZIKV infections can cause severe congenital syndromes, it is crucial to understand any potential enhancement or protection offered by cross-reactive antibodies [8]. Studying the vertical transmission of ZIKV has posed some challenges to researchers. Results produced in mouse models are difficult to translate directly to a human or non-human primate model since mice placentas are structurally different [25,26,27]. Ovine and non-human primate models have proved to be promising, but these too have their limitations, such as increased costs, small sample sizes, and labor intensiveness [28,29]. To address these roadblocks, recent advances in biotechnology have generated co-culture models that use primary human cell lines and stem cells to replicate cellular interfaces. Co-culture models have been used to simulate the blood–brain barrier, the pulmonary barrier, and the maternal–fetal axis in nanoparticle translocation studies [30,31,32,33,34].

The transwell co-culture model was utilized in this study to determine if the cross-reactivity of YFV antibodies could impact ZIKV pathogenesis in utero during early pregnancy. This in vitro model offers multiple benefits, such as reproducibility and standardization, and excels in simulating the physiological boundary of the maternal–fetal axis [35,36]. Syncytiotrophoblasts and umbilical vein cells (BeWo and HUVEC) were used in our transwell co-culture, following established placental models [34,35,37]. The BeWo cell line was derived from a human placenta and best simulates the structure and function of the syncytiotrophoblasts layer of the placenta that forms the continuous outer layer of the placenta [38,39]. These cells directly contact maternal blood and regulate the exchange of nutrients and particles to a developing fetus [25,40,41]. Any virus or antibodies moving across the placental barrier would first have to cross the syncytiotrophoblast layer to reach a fetus, and previous studies have determined the translocation rates across a BeWo layer to replicate the rates found in ex vivo placental perfusions [27,42]. Furthermore, we employed a ZIKV isolate derived from human placenta that we felt would be more relevant than utilizing a lab-adapted strain with unknown tissue tropism in humans.

**Figure 1:**
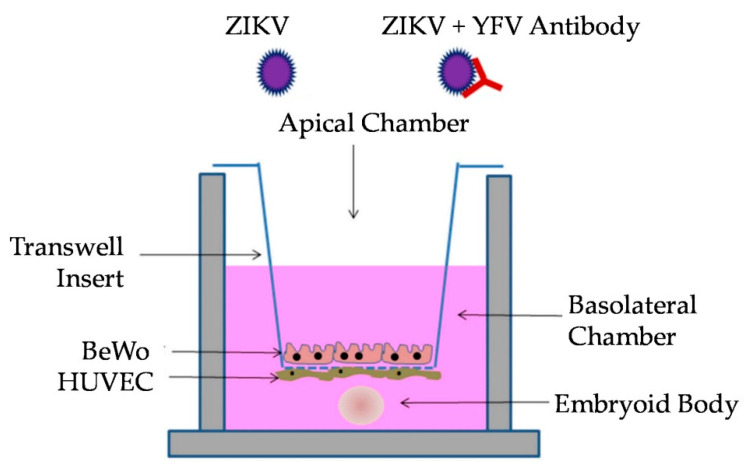
Experimental model of the transwell co-culture assay modified from Campagnolo et al. (2018) [37]. Co-cultures of BeWo, HUVECs, and embryoid body (EB) were apically infected with either Zika virus (ZIKV) or ZIKV+YFV (yellow fever virus) antibody.

The paper by Campagnolo et al. (2018) was followed by including a differentiating embryoid body (EB) in the basolateral chamber of the transwell co-culture, which mimics an early-stage developing embryo [37]. An EB is generated by inducing stem cells to differentiate and self-organize in the three germ layers: the endoderm, mesoderm, and ectoderm [43,44,45]. By including an EB in the co-culture model, the goal was to determine if there were differences in the translocation of the virus and/or virus–antibody complexes that cross the placental barrier and infect an EB. Here, a co-culture model (Figure 1) is described, which can be utilized to study the enhancement or neutralization of a virus by maternal antibodies at the maternal–fetal axis. This study showed that YFV post-vaccination antibodies can enhance the ZIKV infection of an EB, which could impact the development of congenital syndromes.

## 2. Materials and Methods

### 2.1. Cell Culture and Virus Propagation

Primary human umbilical vein endothelial cells (HUVECs) (ATCC PCS-100-013)—normal, human, and pooled—were cultured in EndoGRO-MV-VEGF media (MilliporeSigma, Burlington, MA, USA) containing 5% fetal bovine serum (FBS). To promote the microvasculature phenotypes commonly expressed in the first trimester with placental expansion and throughout pregnancy, a variety of factors were used including rh VEGF, rh EGF, rh FGF, rh IGF, ascorbic acid, hydrocortisone hemisuccinate, heparin sulfate, and 1× Glutamax according to the manufacturers’ instructions [35,46]. Additionally, human placental cells BeWo (ATCC CCL-98) were cultured in Ham’s F-12K (Kaighn’s) Medium containing 10% FBS, 1× non-essential amino acids, 1× Glutamax, and 1 mM HEPES. Lastly, the *Cercopithecus aethiops* kidney cell line Vero E6 (ATCC CRL-1586) was grown in Dulbecco’s modified Eagle’s medium (DMEM) with 10% FBS, supplemented with penicillin/streptomycin, 1× non-essential amino acids, 1× Glutamax, and 1 mM HEPES. All the cell lines were incubated at 37 °C under 5% CO_2_. ZIKV R103451 and YFV 17D were obtained from BEI Resources (Cat. # NR-50355 and NR-116) and expanded once in Vero cells. The virus was titrated using a standard plaque assay in Vero cells as described elsewhere [47]. All the yellow fever antisera were obtained through BEI Resources, and three sera taken from different non-human primates were pooled to account for differential host-specific immune responses (BEIresources.org) [48]. Pre-Immune Yellow Fever Virus Antisera (Cat #NR-42564, primate #08R0137; Cat #NR-42556, primate #067005; and Cat #NR-42565, primate #80485) were taken from non-human primates prior to immunization, Early Immune Yellow Fever Virus Antisera (Cat #NR-29335, primate #067005; Cat #NR-29337, primate #08R0137; and Cat #NR-29338, primate #80485) were collected from non-human primates 30 days post-vaccination with a live attenuated yellow fever virus vaccine (17D), and lastly, Late-Immune Yellow Fever Virus Antisera (Cat #NR-42567, primate #067005; Cat #NR-42575, primate #08R0137; and Cat #NR-42576, primate #80485) were collected from non-human primates at 30-day intervals between 120 and 420 days post-vaccination with a live attenuated yellow fever virus vaccine (17D) and then pooled.

### 2.2. Embryoid Body Formation and Imaging

Human Induced Pluripotent Stem Cells (hiPSC) (WiCell MIN12i-33362.C) were cultured in mTeSR1 medium (StemCell Technologies, Vancouver, BC, Canada) on plates coated with vitronectin XF (Stemcell Technologies, Vancouver, BC, Canada). The hiPSC were dissociated with a gentle cell dissociation reagent and washed with 10 mL of EB formation medium (StemCell Cat#05893) with 10 µM Y-27632 (Stemcell # 72302). Then, 100 µL of undifferentiated MIN12i-33362.C cells were seeded in each well of a 96-well, round-bottom, ultra-low-attachment plate at a density of 90,000 cells/mL in EB medium with 10 µM Y-27632 using a multichannel pipettor to ensure uniformity. The 96-well plate was incubated at 37 °C under 5% CO_2_ for 48 h without being disturbed. On Day Two and Day Four of formation, 100 µL of EB formation medium was gently changed in each well. On Day Five, the EBs were observed for uniformity, each with a diameter between 400 and 600 µm, and smooth round edges prior to being harvested.

In order to visualize the ZIKV infection of the EBs, the EBs were rinsed in PBS, fixed with paraformaldehyde solution at 4% in phosphate buffered saline (PBS) (ThermoScientific CAT# J19943-K2), and blocked in 5% lamb serum. PBS with 0.5% Tween20 was used as a permeabilizing agent. Monoclonal Anti-Flavivirus Group Antigen, Clone D1-4G2-4-15 (BEIresources NR-50327) was used to identify virus, and the detection was conducted overnight at 4 °C. The EBs were rinsed in PBS and then incubated with secondary antibodies at room temperature for 1 h. AlexaFluor 647 goat anti-mouse was used as a secondary antibody (Invitrogen #A21235). The EBs were rinsed again and then placed on slides and mounted in ProLong Gold Antifade Reagent with DAPI (Cell Signaling Technology, Danvers, MA, USA, catalog #8961S). Cover slips were placed on the slides and then gently pressed down to flatten the EB. The slides were incubated overnight at 4 °C. Images were taken with an Olympus Fluoview 3000 confocal microscope and processed using the Olympus Fluoview FV10-ASW 4.1 software package. All images were obtained on the same day using the same imaging parameters (zoom, gain, offset, slices, etc.).

### 2.3. Monolayer Infection and Imaging

Prior to proceeding with the co-culture assay, we verified the permissiveness of BeWo and HUVEC cells to our ZIKV isolate. Confluent BeWo and HUVEC monolayers were infected with 1000 plaque-forming units per well in a 12-well plate. After 48 h, the samples were fixed with paraformaldehyde solution, 4%, in phosphate buffered saline (PBS) (ThermoScientific CAT# J19943-K2) and blocked in 5% lamb serum. Primary antibody staining with an anti-flavivirus-group antigen, Clone D1-4G2-4-15 (BEIresources NR-50327), was performed to visualize ZIKV. The staining was conducted overnight at 4 °C. The cells were rinsed in PBS and then incubated with secondary antibodies at room temperature for 1 h. The secondary antibodies included AlexaFluor 647 goat anti-mouse (Invitrogen #A21235). The cells were rinsed, and then, coverslips were mounted with ProLong Gold Antifade Reagent with DAPI (Cell Signaling Technology, Danvers, MA, USA, catalog #8961S) and incubated overnight at 4 °C. Images were taken with an Olympus Fluoview 3000 confocal microscope and processed using the Olympus Fluoview FV10-ASW 4.1 software package.

### 2.4. ELISA

The presence of IgA, IgG, and IgM antibodies was measured via ELISA. Cell lysates and culture supernatants of Vero cells infected with either ZIKV or YFV-17D were used as the test antigens. Cell lysates and culture supernatants of Vero were used as the mock antigens. This assay was based on previous studies by LaBeaud et al., 2008 [35]. Monoclonal Anti-Flavivirus Group Antigen, Clone D1-4G2-4-15 (BEI #NR-50327), was used as a positive control for both YFV and ZIKV. The secondary antibody, goat anti-monkey IgG conjugated with horse radish peroxidase (Novus Biologicals #NB7215) or goat anti-monkey IgA, IgM, or IgG conjugated with Texas Red (Novus Biologicals #NBP1-73562), was added at a 1:2000 dilution and incubated at 37 °C for 60 min. Anti-monkey IgM is currently on back order from all suppliers, so we used an antibody that simultaneously detected IgA, IgM, and IgG instead. Alexafluor 488 goat anti-mouse was used to detect D1-4G2-4-15. Briefly, YFV antiserum from 3 non-human primates was pooled for each treatment. The serum was diluted 1:100 and read at 405 or 488 nm according to the secondary antibody. The assay plates were normalized to the negative control (DMEM only). Wells with optical density readings were considered positive if they were at least 2 standard deviations greater than the average for the mock antigen after normalization to the average negative control readings.

#### Cross-Reactivity Assay

In order to determine if YFV antiserum had enhancement or neutralization properties for the ZIKV used in this study, a cross-reactivity assay was performed. Virus was incubated with post-vaccination antiserum at a 1:100 dilution with ZIKV in PBS for 60 min at 37 °C. This mixture was then used to inoculate confluent Vero cells (1000 plaque-forming units per well), incubated for an hour at 37 °C, and then overlaid with growth medium in 0.5% methylcellulose. Staining occurred after 5 days as described below.

### 2.5. Embryoid Body Antibody Assay

To gauge the permissiveness of embryoid bodies to ZIKV and the impact of YFV antiserum on the ZIKV infection of EBs, an assay was performed to measure cross-reactivity. Harvested EBs, five days after formation, were placed in a 96-well, round-bottom, ultra-low-attachment plate with fresh EB formation media, with one EB per well. Pooled yellow fever antisera for the Pre-Immune, Early, and Late samples were incubated with 10,000 plaque-forming units of ZIKV at a 1:100 dilution at 37 °C under 5% CO_2_ for 1 h. Each EB was then inoculated with the virus/antibody mixture and incubated at 37 °C under 5% CO_2_ for 72 h. At 72 h, the EBs were disrupted by vigorous trituration and homogenized with the supernatant. ZIKV was then quantified via plaque titration on Vero cells as described below. ANOVA with a Tukey–Kramer post-hoc test for pairwise comparisons and a Shapiro–Wilk test for normality of distribution were performed.

### 2.6. Transwell Co-Culture

Methods previously described elsewhere were followed [35,47]. Briefly, Corning 12 mm Transwell^®^ 3.0 µm Pore PTFE Membrane Inserts (Corning, NY, USA catalog# 3494) were incubated with Matrigel at a 5× coating dilution for 1 h at 37 °C. The inserts were rinsed with DMEM-F12, and then, HUVEC cells were seeded on the basolateral side of the insert at a concentration of 1.0 × 10^5^ cells per 100 µL. The HUVEC monolayer on the basolateral side was achieved using methods described by Aengenheister et al. (2018) [47]. Briefly, the inserts were inverted into 6-well plates, with 1 mL of PBS in one well, to ensure sufficient humidity. Rubber spacers (approximately 1.5 mm thick) were placed on the corner of the 6-well plate to lift the lid slightly and prevent the direct contact of the lid with the inverted insert. After the basolateral side was seeded with HUVECs and the lid was replaced, there was slight adhesion between the lid and the medium. The HUVEC-seeded inserts were then incubated at 37 °C under 5% CO_2_ for 2 h, and afterwards, the inserts were placed back into the 12-well plate containing fresh HUVEC medium. After an insert was replaced in the 12-well plate, the apical layer of the membrane was seeded with BeWo cells at a density of 1.5 × 10^5^ cells per 500 µL. The co-cultures were incubated, with the medium being changed every 48 h, until a 100% confluent layer was observed.

### 2.7. Transwell Antibody Assay

**Figure 2:**
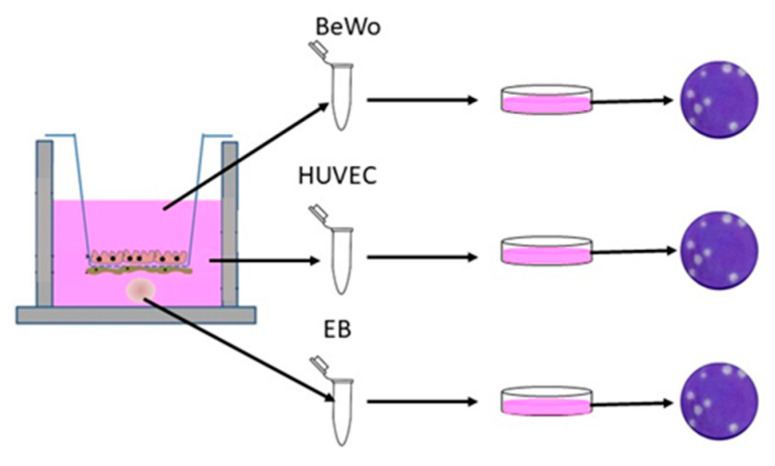
Transwell neutralization assay. Supernatant from BeWo and HUVEC cells was taken at 72 h post-infection (p.i.), and all 3 replicates per cell line were pooled to obtain a single combined solution, which was titrated on Vero E6 cells. EBs were separated from the HUVEC medium and washed in PBS. Three EBs from each replicate were pooled for a total of 9 EBs per treatment for each independent trial. This figure was modified based on the original illustration by Campagnolo et al., 2018 [37].

Prior to infection, the medium in each basolateral chamber was replaced with 1/2 HUVEC medium and 1/2 EB formation medium. Three EBs were added to the bottom of each well using wide-bore pipette tips (Fisher Scientific, Cat. #2079G) to ensure that the EBs were not disrupted (Figure 1). Treatments using YFV antiserum were performed using a 1:100 dilution of pooled serum with ZIKV and were incubated for 1 h at 37 °C. BeWo cells were then inoculated with 100 µL of the mixture (Multiplicity of Infection of 1) in the apical chamber. The assay controls included treatments of mock infection with PBS, Pre-Immune antiserum, Early-Immune antiserum, Late-Immune antiserum, and virus only. To obtain neutralization data, supernatant from BeWo and HUVEC cells was taken at 72 h post-inoculation (p.i.), and all 3 replicates per cell line were pooled to obtain a single combined solution, which was titrated on Vero E6 cells (Figure 2). Samples obtained at 24 and 48 h did not produce quantifiable virus from the EBs or HUVEC cells. The EBs were separated from the HUVEC supernatant by centrifugation at 400× *g* for 5 min. The supernatant was aspirated, and the EBs were washed with PBS before a second centrifugation at 400× *g* for 5 min, after which they were gently resuspended in PBS. Three EBs from each replicate were pooled for a total of 9 EBs per treatment for each independent trial. The results of the titration assay are expressed as the averages of three independent trials with three replicates for each treatment. ANOVA with a Tukey–Kramer post-hoc test for pairwise comparisons and a Shapiro–Wilk test for normality of distribution were performed.

### 2.8. Viral Quantification

Plaque assays were performed using pooled supernatant samples from each transwell treatment following a method described previously [48]. The EBs were separated as described above and vigorously triturated to disassociate the cells. Briefly, serial dilutions of the culture supernatant or EBs in PBS were used to inoculate confluent Vero E6 cells and covered with a 0.5% methylcellulose overlay. The overlay was removed after five days, and the cells were fixed and stained with 5% acetic acid, 43% ethanol, 50% methanol, and 0.2% wt/vol Coomassie Brilliant Blue R-250 prior to counting plaques.

## 3. Results

### 3.1. Placental Cells Are Permissive to ZIKV Infection

**Figure 3:**
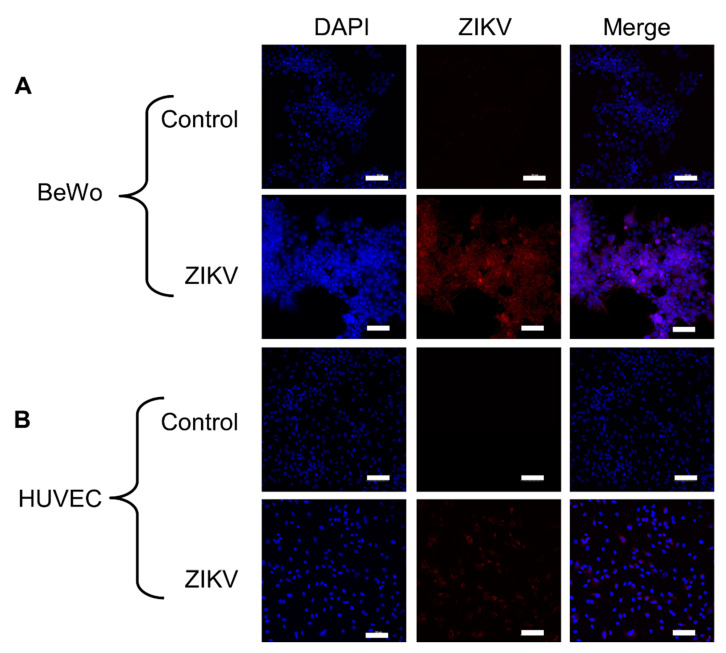
Monolayer infection of BeWo and HUVEC cells with ZIKV viewed under 20× magnification. (**A**) BeWo monolayer stained 48 h p.i. with ZIKV. (**B**) HUVEC monolayer stained 48 h p.i. (blue = DAPI, red = ZIKV–4G2). Scale bar represents 100 µM.

Clinical reports indicate that placental pathology is minimal for ZIKV congenital infections and primarily involves reduced placental weight [49,50,51]. Reports have shown that ZIKV causes autophagy, apoptosis, and other forms of cytopathic effects (CPE) in HUVEC cells, which is contrary to clinical data [52,53]. ZIKV has also been detected in BeWo cells, but investigations regarding CPE are limited [54,55]. Furthermore, these studies did not employ the placental isolate used here, which was derived from the placenta of an infant with microcephaly, which could impact the observations [56]. The purpose of this experiment was to determine if HUVEC and BeWo cells were permissive to our ZIKV strain and if it would not induce excessive CPE that might interfere with the experiment. Monolayers of both BeWo and HUVEC cell lines were infected with ZIKV, and infection was visualized using the anti-ZIKV D1-4G2-4-15 antibody. Staining determined that both the BeWo and HUVEC cell lines are permissive to ZIKV infection (Figure 3). This aligns with previous research showing placental trophoblasts and endothelial cells to be permissive to ZIKV infection [55,57,58,59]. There were no noticeable cytopathic effects within the BeWo and HUVEC cell lines when infected with ZIKV (Figure 3).

### 3.2. YFV Post-Vaccination Antiserum Is Cross-Reactive with ZIKV Antigen

ELISA to identify the IgM, IgA, and IgG serum composition and viral cross-reactivity was performed. Plates were coated with either YFV or ZIKV antigen, and then, IgG, IgA, and IgM were measured. All the control, Pre-Vaccination and Early antisera were negative for both YFV and ZIKV antigens. IgM was not identified in any of the sera on either YFV or ZIKV. IgG was identified in the Late antiserum for both ZIKV and YFV antigens. The anti-IgA, IgM, and IgG antibodies gave a positive reading for only the Late antiserum on YFV antigen (Table 1).

ELISA was used to determine if the translocation of antibodies across the transwell had occurred at 60 min and 72 h following inoculation. Culture medium was directly plated onto antigen without dilution. All the samples obtained 60 min p.i., data not shown, were negative for antibodies. The readings taken 72 h p.i. were positive for IgG for BeWo cells only with the Late antiserum.

### 3.3. Late YFV Post-Vaccination Antiserum Enhances ZIKV In Vero Cells

**Figure 4:**
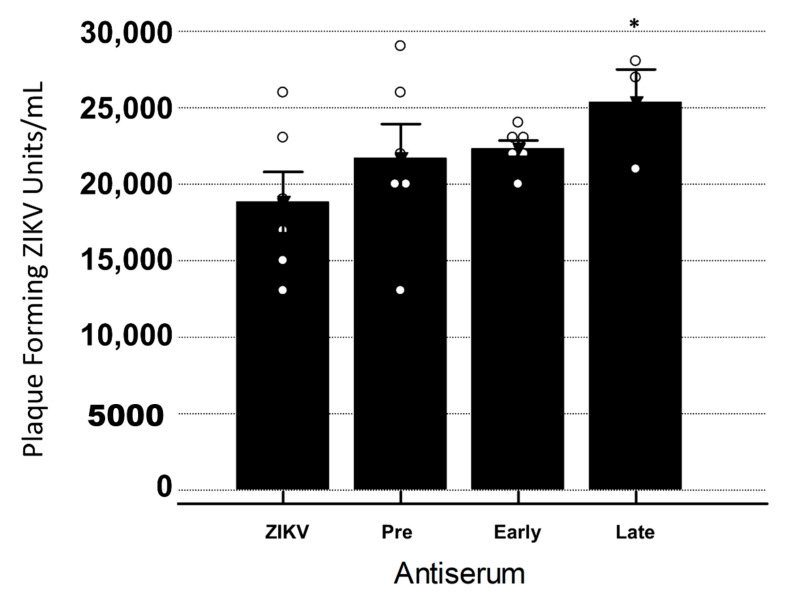
YFV antiserum neutralization/enhancement of ZIKV in Vero cells. Bars signify average plaque-forming units per mL ± standard error. Dots represent individual data points for the treatment. ANOVA with a Tukey–Kramer post-hoc test for pairwise comparisons and a Shapiro–Wilk test for normality of distribution were performed. Significant increases in ZIKV titers were seen when Late YFV antiserum was present when compared to ZIKV only (*p* < 0.001). Statistical significance is denoted by *. All ° are individual data points within that group.

In order to determine if YFV antiserum was cross-reactive in vitro, a standard neutralization assay was performed. Virus was incubated with antiserum and then used to inoculate Vero cells and titrated. An analysis of variance indicated no significant interaction between the treatments, while post-hoc pairwise comparisons indicated that the viral titers were significantly greater when the Late antiserum was present with ZIKV as compared to ZIKV only (*p* < 0.001) (Figure 4).

### 3.4. YFV Early Antiserum Enhances ZIKV in Embryoid Bodies

**Figure 5:**
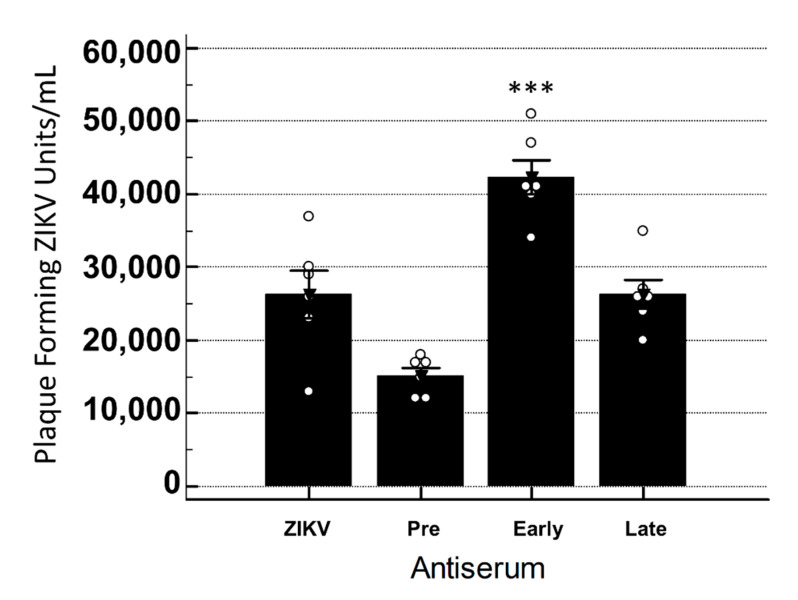
Viral titration of EBs after direct infection with ZIKV or ZIKV + Antisera. Bars signify average plaque-forming units per mL ± standard error of 3 independent trials with 3 replicates each. Dots represent individual data points for the treatment. ANOVA with a Tukey–Kramer post-hoc test for pairwise comparisons and a Shapiro–Wilk test for normality of distribution were performed. Significant increases in ZIKV titers were seen in EBs when Early antiserum was present as compared to ZIKV only (*p* < 0.001). Statistical significance is denoted by ***. All ° are individual data points within that group.

In order to identify if the antisera could directly impact the viral infection of the EB, the antiserum was incubated with ZIKV for 1 h at 37 °C and then used to inoculate the EBs. Viral titers were obtained 72 h post-inoculation. When treated with the Early antiserum, the EBs produced significantly more virus than with ZIKV alone (*p* = 0.0008) (Figure 5). The other antisera did not cause significant changes in the ZIKV titer when present as compared to ZIKV only (*p* < 0.001) (Figure 5).

### 3.5. YFV Late Antiserum Reduces ZIKV Infection of EBs in Co-Culture

**Figure 6:**
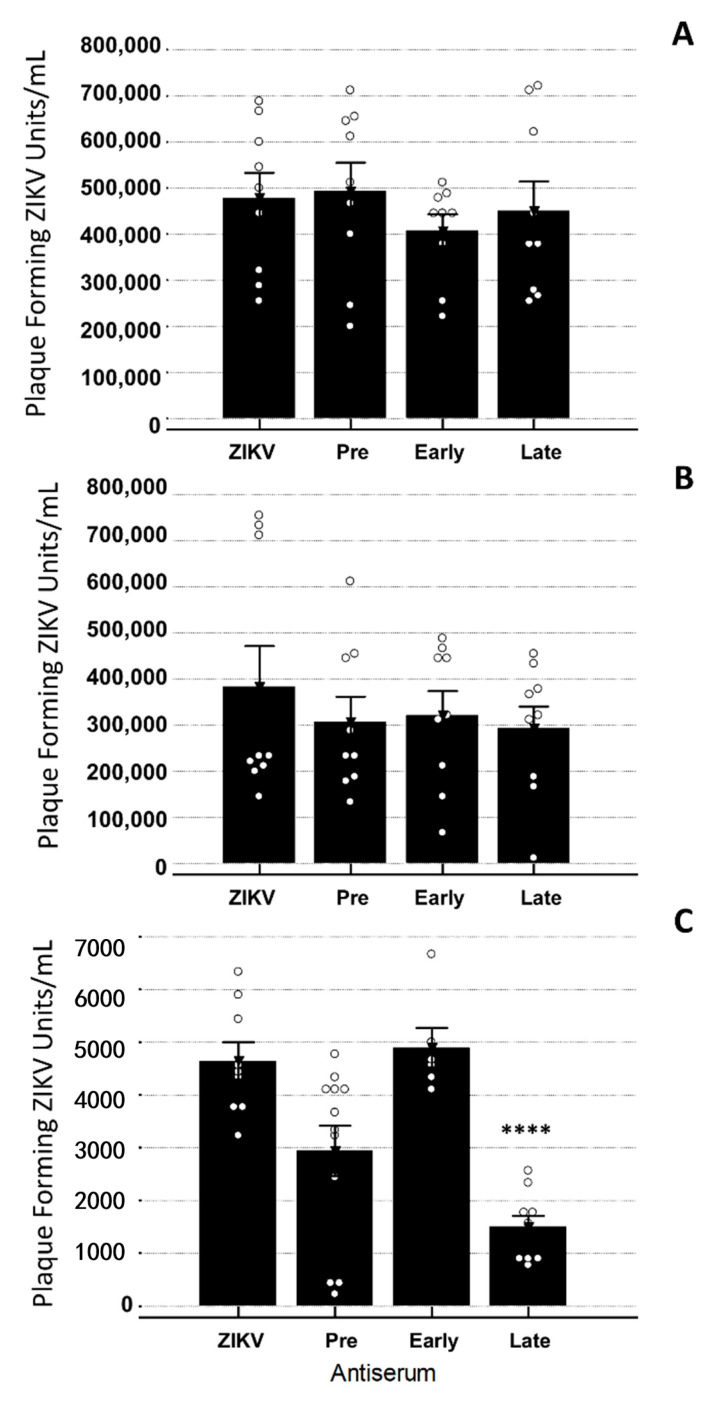
Viral titration of ZIKV in BeWo, HUVECs, and EBs from transwell co-culture 72 h p.i. Bars signify average plaque-forming units per mL ± standard error of 3 independent trials with 3 replicates each. Dots represent individual data points for the treatment. ANOVA with a Tukey–Kramer post-hoc test for pairwise comparisons and a Shapiro–Wilk test for normality of distribution were performed. (**A**) There were no significant changes in ZIKV titer in BeWos from the transwell co-culture when YFV antisera were present. (**B**) HUVEC cells from the transwell co-culture exhibited no significant difference in ZIKV titer when YFV antisera were present. (**C**) Significant decreases in ZIKV titers were seen in EBs taken from the transwell co-culture when Late YFV antiserum was present, when compared to ZIKV only (*p* < 0.001). Statistical significance is denoted by ****. All ° are individual data points within that group.

**Figure 7:**
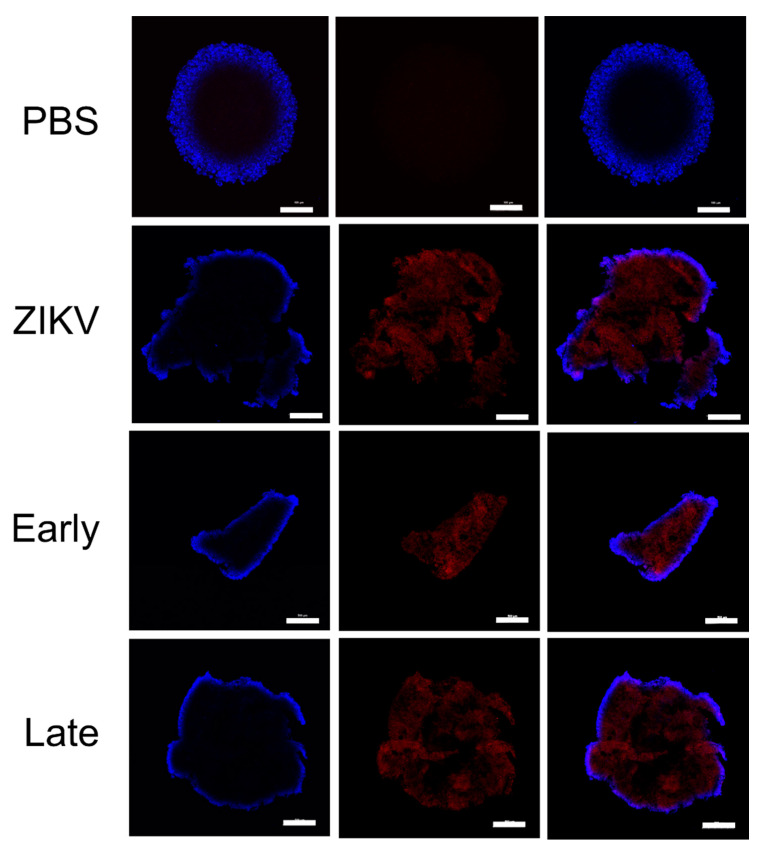
Immunofluorescence of EBs (blue = DAPI, red = ZIKV–4G2). EBs were stained with D1-4G2-4-15 antibody and mounted on glass slides. Coverslips were pressed down on the EBs to flatten them, producing a ring effect in most images. Images were obtained under 4× magnification. Scale bar represents 100 µM.

In order to determine if the translocation of virus across the transwell had occurred, samples were obtained 60 min following inoculation. Culture medium from both sides of the transwell was titrated on Vero cells. All the samples were negative for viral plaques (data not shown). At 72 h p.i., ZIKV was detected via the viral plaque assay. The presence of YFV antibodies, regardless of the time the antiserum was taken post-vaccination, did not have any significant impact on ZIKV viral titers in the HUVEC or BeWo cell lines (Figure 6). The EBs had significantly decreased levels of infectious ZIKV at 72 h p.i. when the Late YFV antiserum was present when compared to ZIKV alone (*p* = 0.000006) (Figure 6). Imaging studies of the EB verified that ZIKV and CPE could be detected throughout the EB for all the treatments except the non-virus control (Figure 7). The variation in size observed reflects the normal variance of the size when EBs are generated in the manner we employed [60].

## 4. Discussion

Here, data are presented showing that ZIKV derived from the human placenta can infect both the BeWo and HUVEC cell lines and replicate without causing excessive CPE. Other studies have documented CPE in ZIKV-infected placental cell lines, which could be due to experimental differences in the cell lines and virus strains [11,61]. ZIKV has been known to infect placental tissue in humans as well as in animal models, and it can cross the placental barrier to infect a developing fetus to cause congenital syndromes [11,28,62,63,64]. The data in this study show that within 72 h p.i., ZIKV effectively crosses two monolayers, a basement membrane, and infects an EB. The detection of ZIKV in cells located on each side of the membrane as a model for the maternal–fetal axis supports reports of the isolation of ZIKV from placentas and fetuses [28,50,65,66]. However, the permissiveness of the cells observed in this study may not reflect the cellular tropism of ZIKV in actual placentas [67,68,69,70].

Of interest, the interactions of the YFV antisera varied depending on the cell line infected. The addition of the Late antiserum resulted in ZIKV neutralization in the co-culture model but did not neutralize when the EBs were directly infected. Further, the Early antiserum enhanced ZIKV infection when the EBs were directly infected but not in the co-culture model. When Vero cells were infected with virus/antiserum, the ANOVA results were not significant, which agrees with other research where YFV/ZIKV cross-reactivity was explored [71,72,73].

The Pre-Vaccination serum was collected prior to vaccination with the 17D YFV vaccine, the Early serum was collected 30 days p.i., and the Late serum was collected at 30-day intervals between 120 and 420 days p.i. It has been shown that broadly neutralizing IgM antibodies quickly appear after vaccination with the 17D vaccine, typically 4–7 days p.i., and have been found to circulate anywhere between 2 and 11 years p.i. [74,75,76]. Other research has shown that the IgM response is donor-specific, with antibody maturation taking 6–9 months post-vaccination to complete [77]. Wec et al. also showed that classical IgM waned rapidly over time, while swig + IgM were stable for at least a year post-vaccination [77]. It could be that the secondary antibody used in our studies did not detect swig + IgM. While IgM was not detected in any of the antisera, enhancement occurred when the EBs were directly infected with serum collected 30 days post-vaccination. Young IgG antibodies tend to exhibit increased cross-reactivity in relation to older IgG antibodies and could explain the enhancement we observed when the EBs were directly infected [78,79]. It is possible that some of the phenotypic effects observed were due to serum factors that are not necessarily altered by vaccination.

These differences are important to consider when analyzing why some sera in this study enhanced or neutralized while others did not. IgG antibodies have Fc regions and can readily cross the placenta due to the FcRn receptor on placental syncytiotrophoblasts and endothelial cells and have already been found to be endocytosed by the BeWo cell line [80,81,82,83]. By the time Late antiserum was collected, IgG antibodies would have been abundant, thus suggesting why ZIKV neutralization was observed in the co-cultured EBs while enhancement was observed in the Vero cells and directly infected EBs.

Anti-DENV antibodies have been shown to enhance ZIKV infection in multiple model systems and case studies [9,11,83,84,85]. Due to the genetic similarities between flaviviruses, it would be expected that antibodies for other flaviviruses, such as YFV, may also have the ability to enhance or neutralize ZIKV infection. In this study, the EBs from co-cultures had lower viral loads as compared to the placental cell lines. This supports reports of ZIKV titers in fetal and placental tissues in vivo [28,65,66]. Since macrophages or other monocytes were not used in this study, the enhancement we observed was likely due to antibody-mediated enhancement (AME). AME occurs when antibodies bind to virus particles, forming complexes. These complexes interact with cell surface receptors and promote entry into host cells, leading to increased levels of viral replication via the suppression of innate immune processes and inflammatory cascades [24,86]. While this process is associated with Fc receptor-bearing monocytes, it is also possible for these virus–antibody complexes to infect other cell types and suppress innate immunity [24].

The data presented here highlight the impact of various cells on the neutralization or enhancement of ZIKV with YFV antisera. The cell-specific responses of ZIKV and other flaviviruses is well documented and should be recognized when developing cross-reactivity assays [48,87]. The data also highlight the impact of placental cells and maternal antibodies on the establishment of congenital ZIKV infections. A limitation of these studies was the unavailability of anti-monkey IgM secondary antibody. For this research, we utilized a secondary antibody that detects IgA, IgM, and IgG. This secondary antibody detected IgG antibody but not IgM. Whether this was due to a lack of sensitivity of the antibody or the lack of IgM in the antisera is unknown.

A second limitation of this study was the omission of Hofbauer cells from the model. Hofbauer cells have been found to play a role in ZIKV transmission through the placental barrier [83,88,89,90]. They are placental macrophages that also have Fc receptors, play a role in early angiogenesis within trophoblast cells, and have been found to transfer viral particles into the fetal endothelial cells and blood supply [81,91,92,93]. Since they have been found to further facilitate the vertical transmission of ZIKV, not including them in the transwell co-culture assay limited the findings within this study. DENV antibodies have already been found to enhance ZIKV infection within Hofbauer cells, and as such, not including them could have impacted the results [83]. We did not include Hofbauer cells in this assay since they are primary cells that have strict culture requirements that were not compatible with the other cells in this assay.

## 5. Conclusions

Due to the already-established cross-reactive nature of flavivirus antibodies, it is crucial to understand the interactions of neutralizing and enhancing antibodies as vaccine research continues for many of these viruses [9,10,94,95]. The 17D vaccine for YFV has been included in the recommended vaccinations in most Latin American countries, and ongoing vaccination campaigns are vaccinating more adults, creating a population of people with potentially cross-reactive antibodies [4,5,6,12,75]. Additionally, in regions of Latin America where these viruses co-circulate due to a common vector, *Aedes* mosquitos, many people have the potential to become co-infected or infected by different flaviviruses within their lifetime [1,4,8,89]. Further research about the cross-reactive nature of flavivirus antibodies in co-culture models is needed, especially because vertically transmitted viral infections can lead to congenital syndromes. More studies are also needed to better understand the kinetics of antibody passage through the transwell co-culture model, and to better understand the possible antibody-mediated enhancement of ZIKV by YFV antibodies produced after receiving the 17D vaccine, especially in pregnant women who may be receiving a booster or receiving vaccination during campaigns.

## Figures and Tables

**Table 1:** Antibodies detected in post-vaccination antisera. Antibodies were bound to either YFV or ZIKV antigen. Secondary antibodies consisted of goat anti-monkey IgG, or IgA, IgM, and IgG.

ELISA	YFV Antigen			ZIKV Antigen		
	Pre	Early	Late	Pre	Early	Late
IgA, IgM, IgG	-	-	+	-	-	-
IgG	-	-	+	-	-	+
